# Treatment-Refractory, Castration-Resistant Prostate Cancer With Liver Metastasis: A Promising Modality of Therapy

**DOI:** 10.7759/cureus.26881

**Published:** 2022-07-15

**Authors:** Dawood Findakly, Tony Duong, Tim Shimon, Jue Wang

**Affiliations:** 1 Hematology and Medical Oncology, Feist-Weiller Cancer Center, Louisiana State University Health Shreveport, Shreveport, USA; 2 Internal Medicine, University of Arizona College of Medicine, Phoenix, USA; 3 Internal Medicine, Creighton University School of Medicine, Phoenix, USA; 4 Genitourinary Oncology, Dignity Health Cancer Institute, St. Joseph’s Hospital and Medical Center, Phoenix, USA

**Keywords:** castration-resistant metastatic prostate cancer, interventional radiology, percutaneous microwave ablation, poly (adp-ribose) polymerase-1 (parp1) inhibitor, liver metastasis

## Abstract

Although significant advances in the treatment of prostate cancer (PC) have recently been made, the treatment of metastatic liver disease remains challenging. Recent advances have led to multiple novel therapies and multi-treatment approaches combining systemic and locoregional modalities, such as thermal ablation, representing a promising strategy that has received attention in recent years. Nevertheless, no standard locoregional treatment regimens exist for the management of liver metastases of PC. In addition, regional therapy alone is unlikely to provide durable cancer control. Here, we report for the first time a successful treatment of hepatic metastases of PC using stereotactic image-guided percutaneous microwave ablation and the poly (ADP-ribose) polymerase-1 inhibitor, olaparib.

## Introduction

Prostate cancer (PC) is the second leading cause of death from cancer among men in the United States. Hepatic metastases in PC are very rare and remain poorly understood [1,2]. Treatment options for patients with metastatic castration-resistant PC (mCRPC) with liver metastasis are limited given the terminal disease at the time of diagnosis and the inherently aggressive nature of the disease at this stage. In this report, we present the case of a 77-year-old man with mCRPC who sustained remission after receiving percutaneous microwave ablation (MWA) and olaparib therapy.

## Case presentation

**Figure 1:**
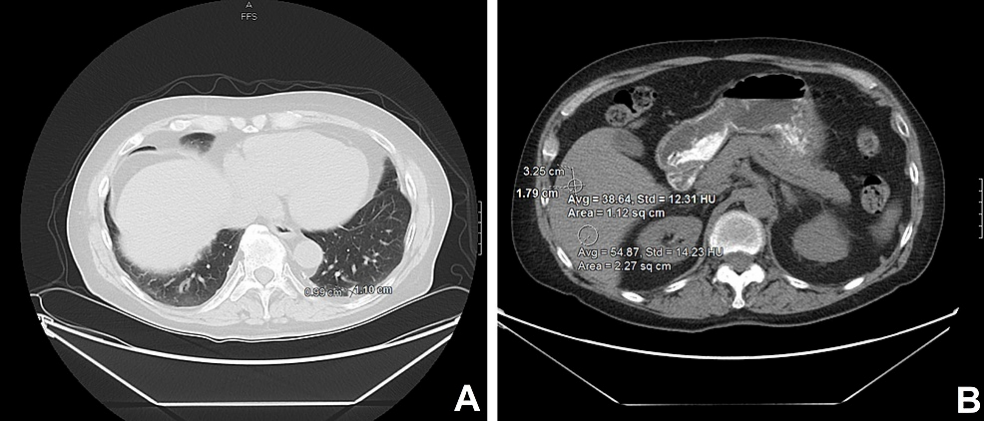
Axial CECT scan of the chest, abdomen, and pelvis at the time of consultation. The images show (A) a 1.0 × 1.1 cm non-calcified left lower lobe pulmonary nodule and (B) a 1.8 × 3.3 cm right liver lobe geographic hypodense region consistent with metastasis. CECT: contrast-enhanced computed tomography

**Figure 2:**
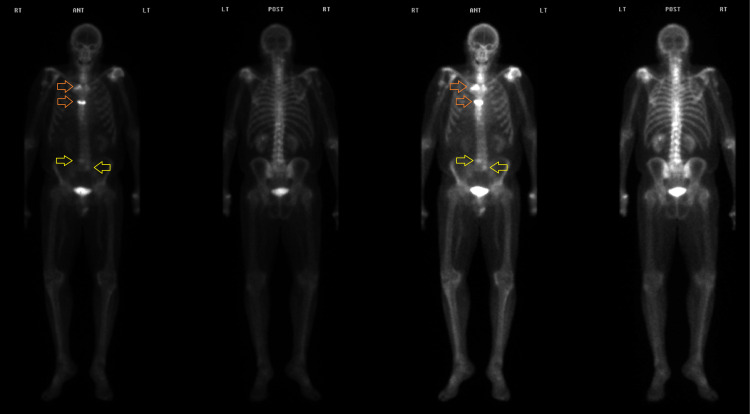
Nuclear bone scan. The images show new areas of mild uptake in the left sacral alae (yellow open arrows) and small focal areas of uptake of the mid and lower thoracic vertebral bodies (orange open arrows), findings concerning metastatic bone lesions.

A 77-year-old Caucasian man presented to our institution seeking consultation for increasing prostate-specific antigen (PSA) level up to 9.1 ng/mL over the past two years despite receiving leuprolide and bicalutamide for his PC with liver metastasis. His condition had started nine years prior when he underwent radical prostatectomy for a Gleason score of 7 (4+3) and T3a N0 M0 PC. Upon this presentation, an axial contrast-enhanced computed tomography (CT) scan revealed a 1.0 × 1.1 cm non-calcified left lower lobe pulmonary nodule and a 1.8 × 3.3 cm right liver lobe hypodense region consistent with hepatic metastasis (Figure 1). Subsequently, a nuclear bone scan revealed new areas of mild uptake in the left sacral alae besides small focal areas of uptake of the mid and lower thoracic vertebral bodies, findings concerning metastatic bone lesions (Figure 2).

The patient was subsequently treated with flutamide and enzalutamide with good PSA response, and the treatment was well tolerated. Six months later, a repeat PSA level was 1.82 ng/mL. The bone scan showed complete remission of bone metastases, but a subsequent CT scan showed an unchanged pulmonary nodule with two hypodense right liver lobe lesions measuring 0.7 × 0.8 cm and 1.7 × 1.2 cm. The patient, thereafter, received degarelix and enzalutamide therapy, but, unfortunately, the PSA levels continued to increase to a level of 4.2 ng/mL despite therapy. Therefore, the tumor tissue was evaluated for specific mutations with next-generation sequencing (NGS). Results of the molecular profiling showed both germ-line mutations in *BRCA2 *and *AR *genes. The tissue revealed a microsatellite stable tumor with a mutational burden (TMB) of 5.3 Muts/Mb. Three months later, the patient had two episodes of hematuria, during which a repeated PSA was found to be 18.7 ng/mL. A CT scan of the abdomen and pelvis showed two enlarging hypoattenuating hepatic lesions measuring 3.3 × 2.5 cm and 1.7 × 1.2 cm, and a repeat bone scan did not suggest any osseous metastatic disease. Subsequent ultrasound-guided core needle biopsy of the hepatic lesion was consistent with prostatic ductal adenocarcinoma.

**Figure 3:**
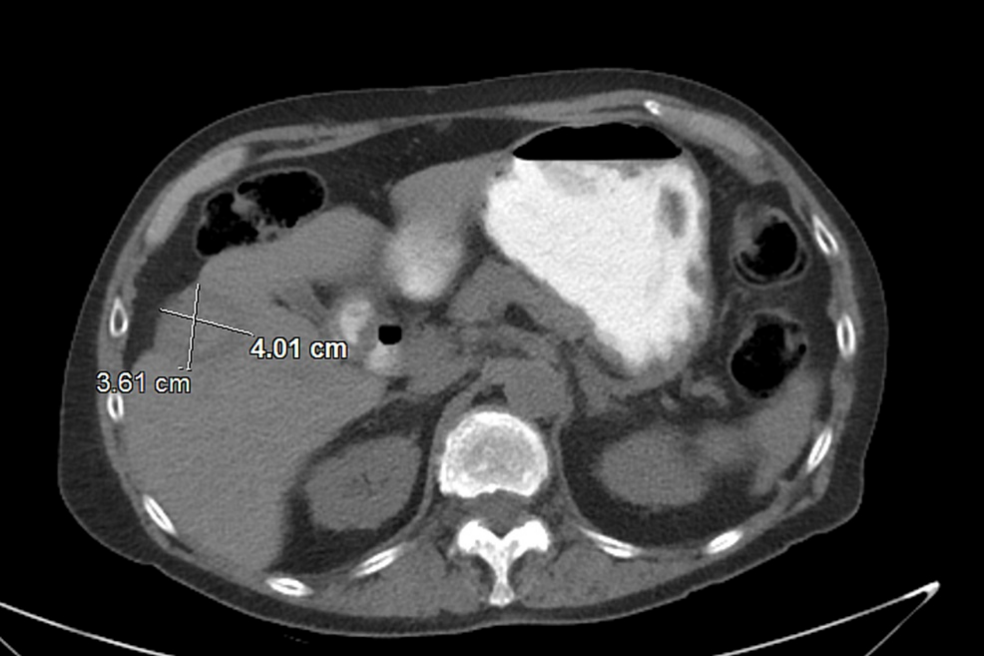
CT scan 48 months after liver metastasis and 18 months after MWA. CT scan showed treatment effects of an ill-defined hepatic mass measuring 4.0 cm without new liver lesions. MWA: microwave ablation, CT: computed tomography

Thereafter, the patient received one cycle of docetaxel and prednisone. A molecular tumor board was convened to discuss his condition. Given poor tolerance to pharmacological systemic therapy, local ablation was considered. Accordingly, he underwent a CT and ultrasound-guided MWA of the liver without complications. The patient’s PSA at the time of the procedure was the highest at 39.5 ng/mL. Based on the findings of *BRCA *which suggested that he may have increased sensitivity to a poly (ADP-ribose) polymerase-1 (PARP1) inhibitor as a potentially actionable alteration, and given his progressive disease despite multiple lines of therapies, he was started on olaparib 400 mg twice-daily oral therapy. The treatment was well tolerated with only occasional mild diarrhea and fatigue. The patient remains alive with complete remission upon repeat imaging at 48 months after the initial diagnosis of liver metastasis (Figure 3).

## Discussion

More than 72% of mCRPC have a predilection to the bone, and only about 4% metastasize to the liver. Hepatic metastasis usually reflects disease aggressiveness with a worst median overall survival compared to other visceral organ metastasis [3,4].

Despite medication development in the past decade, mCRPC remains deadly with limited therapeutic options [5,6]. The majority of approved anticancer drugs for PC, including enzalutamide, abiraterone/prednisone, docetaxel, and cabazitaxel, do not have a durable impact in the setting of established metastatic liver disease due to acquired therapeutic resistance [7].

Olaparib was approved by the Food and Drug Administration for the treatment of mCRPC in mid-2020 [8]. For patients with mCRPC who had disease progression while receiving hormonal therapy and who had alterations in DNA damage repair genes, olaparib was found to be associated with more extended progression-free survival and better response than enzalutamide or abiraterone [9]. In our patient, the combination of percutaneous MWA and olaparib led to an impressive durable treatment response with a significant reduction of hepatic tumor burden fulfilling the response criteria of complete remission.

Combinations of systemic therapy and locoregional modality represent a promising strategy that has received renewed attention in recent years. With the current scarcity of effective treatment options in more advanced disease stages, MWA of the liver may provide a viable treatment option in mCRPC patients with hepatic metastases. However, after an extensive literature search and to the best of our knowledge, there is limited information on the use of ablation systems for the treatment of liver metastases due to PC. Moreover, this is the first case report utilizing MWA techniques in combination with systemic therapy to achieve durable control of hepatic metastases in an elderly patient with mCRPC.

## Conclusions

This case demonstrates that a combined modality may improve outcomes in patients with metastatic mCRPC and adds to a growing body of literature in support of the selective use of MWA as part of a multi-modality strategy in the setting of metastatic disease. We aimed to highlight that local control should be carefully considered in selected patients with mCRPC affecting the liver. Hence, there is a growing need to conduct prospective validation of these findings to help establish optimal patient selection for definitive therapy and potentially improve survival for patients with mCRPC. We plan to conduct a prospective, single-center, phase II clinical study to evaluate the safety and efficacy of MWA in combination with systemic therapy for liver metastasis in patients with mCRPC involving the liver.
